# Bacterial vaginosis (BV) and *Trichomonas vaginalis* (TV) co-infection, and bacterial antibiogram profile of pregnant women studied in Lagos, Nigeria

**DOI:** 10.1186/s12905-024-03257-y

**Published:** 2024-07-23

**Authors:** Christian Azubike Enwuru, Adenike Shola Aiyedobgon, Morakinyo Bamikole Ajayi, Kazeem Adewale Osuolale

**Affiliations:** 1 Centre for Infectious Disease Research, Microbiology Department, Nigerian Institute of Medical Research (NIMR), 6 Edmund Crescent (Off Murtala Mohammed Way) PMB, 2013, Yaba, Lagos, Nigeria; 2 Molecular Biology and Biotechnology Department, Nigerian Institute of Medical Research (NIMR), 6 Edmund Crescent (Off Murtala Mohammed Way) PMB, 2013, Yaba, Lagos, Nigeria; 3 Monitoring & Evaluation Unit, Nigerian Institute of Medical Research (NIMR), 6 Edmund Crescent (Off Murtala Mohammed Way) PMB, 2013, Yaba, Lagos, Nigeria

**Keywords:** Pregnant women, Antenatal, Bacterial vaginosis, Co-infection, *Trichomonas vaginalis*, Antibacterial profile, Nigeria

## Abstract

**Aim:**

This study was undertaken to determine the prevalence of Bacterial Vaginosis (BV), *Trichomonas Vaginalis* (TV) co-infection, and the antibacterial sensitivity profile of bacterial isolates.

**Methods:**

The study was a cross-sectional study of 232 pregnant women on a routine antenatal visit between April 2019 and Sept. 2020, at Amukoko clinic in Lagos, Nigeria. The gynaecologist conducted the clinical examination on each patient looking for vaginal discharge and its consistency/homogeneity, colour and odour. Two High Vaginal Swab (HVS) samples were taken from every patient and a semi-structured questionnaire was used to gather the socio-demographic, practices/attitudes, and clinical information of each participant. One sample was employed for wet preparation to identify the TV and BV diagnosis using Amsel’s criteria and Whiff’s test. The second sample was used for bacterial culture and antibiogram was conducted using the disc diffusion technique. The Clinical Laboratory Standard Institutes’ (CLSI) interpretative criteria were used to categorise the results.

**Results:**

The mean age of the clients was 28.11 ± 7.08 years of age. The majority (88%) were aged 15–35 years. Only 81 (34.9%) had microbial organisms isolated or seen from their specimens and 19 (8.2%) of such were classified as having BV (Bacteriods or Gardnerella isolated). Of the 81 infected, 33 (40.8%) had only bacterial infection, 36 (44.4%) had TV alone and 12 (14.8%) had bacteria co-infected with TV. From the clinical records, the population that was classified as having UTI or vaginitis was only 46 (20.7%) The study observed age (15–35 years) related association between vaginosis/ TV co-infection (X^2^ = 7.9; *P* = 0.005). Participants with symptoms of vaginitis or UTI (mainly *E. coli* & pseudomonas spp. isolated), BV/co-infection with TV significantly associated with female traders (X^2^ = 8.5; *P* = 0.003) and were more associated with those from polygamous relationships (X^2^ = 18.79, *P* = 0.0001). Women in their 3^rd^ and 2^nd^. trimester were more significantly associated with vaginal infection (X^2^ = 9.47, *P* = 0.002; X^2^ = 4.79, *P* = 0.029) respectively. The Pseudomonas showed susceptibility to ciprofloxacin (CIP) and cefuroxime (CXM). While, *E. coli* isolates were susceptible to cefepime, ciprofloxacin, and imipenem.

**Conclusion:**

There is a relatively low prevalence of BV and flagellate co-infection in the community studied.

**Recommendation:**

We recommend screening of antenatal women with underlying symptoms for BV and flagellates co-infection to avoid its progression to vaginitis.

**Supplementary Information:**

The online version contains supplementary material available at 10.1186/s12905-024-03257-y.

## Introduction

Bacterial vaginosis (BV) which may sometimes be confused with vaginitis is an imbalance of microbiome in a female’s vagina. Vaginosis results when the naturally occurring normal flora of the vagina such as Hydrogen peroxide ( H_2_O_2_) and lactic-acid-producing lactobacilli that help protect the vagina from harmful microbes are eliminated due to exogenous or endogenous factors affecting the vagina [1]. The resultant effect of the micobiota alteration is a change in the P^H^ of the vagina from acidity to alkaline [2]. While vaginitis, on the other hand, is an inflammation or infection of the vagina or the vulva; with clinical presentations ranging from malodour, thrush, itching, burning sensation, abnormal vaginal discharge, pain during sexual intercourse, and light vaginal bleeding or spotting [3]. Etiologic agents of vaginitis are microbes (bacteria, candida species -fungus and flagellates e.g. *T. vaginalis*—a parasite) [3]. Risk factors associated with vaginitis include vaginal sex with lubricants, use of sex toys and antiseptic soap, estrogen deficiency, pregnancy or menopause, and conditions like allergies and use of irritants like scented tampons or certain detergents [3]. In bacterial vaginosis the altered micro ecosystem warrants overpopulation of mainly facultative or anaerobic bacteria like *Gardnerella Vaginalis*, Mobiluncus spp., Prevotella spp., *Atopobium vaginae*, Peptostreptococcus, Bacteroides species, Fusobacterium species, including microbes-like *Ureaplasma urealyticum*, *Mycoplasma hominis* and others [4, 5]. The domination of the microbiota by some of these organisms causes unpleasant symptoms [4]. It is usually not regarded as a sexually transmitted disease (STD) however; the condition can increase the chances of contracting STD [6]. Usually, vaginosis presents with a fishy odour that may intensify after intercourse, a thin grey or white, or greenish discharge, copious itching and vaginal irritation on the volva, and a burning sensation during urination [7]. Risk factors associated with BV are: having a new sex partner or multiple sex partners, douching, use of feminine sprays and washes, and taking long baths with perfumed oils or soaps [8].

It has been reported that 50–75% of women suffering from such conditions are asymptomatic; affecting about a million women aged 15–44 years [8, 9]. In the US, the BV of pregnant women ranges from 5.8–19.3% [4]. Bacterial vaginosis is reported to be the most prevalent cause of vaginal discharge or malodour in pregnant women [10].

Bacterial and parasite co-infection is the simultaneous infection of a host by multiple pathogen species. Global incidence and prevalence of co-infection among pregnant women have not been well studied, but it is thought to be commonplace [11]. Co-infection is important in human health because different pathogens could interact within the host microbiota with deleterious or symbiotic effects on other pathogens or the host [12]. For instance, it has been reported that Trichomoniasis and BV organisms can reliably coexist in a female vagina; both benefiting from the elevated vaginal P^H^ and anaerobic environment created by the eradication of vaginal normal flora [13]. Syndemism and comorbidity of untreated BV co-infected with other pathogens may progress to vaginitis or urethritis [14]. The general patterns of ecological interactions between most pathogens or their hosts are vague, even among common co-infections such as those between sexually transmitted infections [15]. These critical nebulous health conditions have made treatment of BV co-infection with flagellates imperative, using medications indicated, as against empiric therapy, especially on pathogenic bacteria. Several health consequences underscore this position, for instance, epidemiological studies have demonstrated that abnormal vaginal microbial environment and lower genital tract infections are closely associated with an increased risk for human immunodeficiency virus (HIV) infection [16]. These changes result in congestion and hypertrophy of vaginal mucosa, which consequently allows more growth of anaerobic bacteria and other pathogenic microorganisms within the vagina [17].

Moreover, there is hypertrophy of the cervical gland and proliferation of cervical cells which in turn decreases the B-lymphocyte numbers and reduces the local resistance of the cervix and vagina to infectious agents [18]. Increasing opportunity for microbial co-infection leads to inflammation in the vagina and cervix, thereby increasing the risk of foetal or neonatal morbidity and higher perinatal mortality [19]. This is possible because the cervix is usually a barrier to keep microbes from accessing the uterus**.** However, when the cervix is infected, there is a preponderance of risk of uterus infection that may affect the foetus in pregnant women [20].

Again, vaginal dysbiosis during the early stages of pregnancy is gaining recognition due to its positive association with adverse pregnancy outcomes [21]. On the other hand, TV has been described as a common cosmopolitan parasite of both male and female genitals and is sexually transmitted. It is estimated that 174 million new cases are reported in resource-poor countries [22]. Approximately, 180 million people are infected worldwide annually [23]. In Africa (west and central 20.6%, East 33.3%), a median 20% prevalence of women attending gynaecologic clinics had TV, while the prevalence in Asia was reported as 11%, Europe 22.8%, North America 27.4% and Latin America, up to 24.2% [24].

Standard treatment with antibiotics such as metronidazole or tinidazole and clindamycin, with sexual partners is recommended for symptomatic pregnant women [9]. In non-pregnant women, treatment is indicated to gain relief from the vaginal symptoms and reduction in the risk for acquiring *C. trachomatis*, *N. gonorrhoeae*, *T. vaginalis*, *M. genitalium* and viruses (HIV, HPV, and HSV-2) [9]. Reports on the increasing rates (> 60%) of BV recurrence after therapy abound; thereby inducing antimicrobial resistance in BV-associated bacterial infections, including those that could form biofilm in the vaginal canal [5]. Antibacterial resistance is a global phenomenon [22]. Failure to treat BV poses a risk of infertility and in pregnant women may lead to complications such as preterm labour and preterm birth [10, 20, 25]. However, some schools of thought do not support routine screening for BV in asymptomatic pregnant women [4].

In the patients indicated for treatment for bacterial eradication, multiple antibiotic resistances is a great challenge, coupled with the absence of newer antimicrobial agents to treat drug-resistant pathogenic vaginal microbes, therefore, an antibacterial resistance profile is required to make the best drug choice or combination selections as reported by Muzny & Sobel [5].

The study investigated BV co-infection with common vaginal flagellate (TV). The study is justified since BV is reported as the most prevalent cause of vaginal discharge in pregnant women and that co-infection with TV may bring about complications of vaginitis or urethritis and biofilm formation making the infection difficult to treat [5] . There is a paucity of information on BV and flagellate co-infection in Nigeria and their possible complications. However, a report on the significant association of BV in pregnant women with preterm delivery and low birth weight in SW Nigeria was made by Afolabi et al. [26], in a longitudinal study. Therefore, in other to keep track of the current common vaginal health of pregnant women, we studied the BV and TV co-infection in Amukoko, –an urban slum in Lagos, Nigeria.

## Materials and methods

### Study design

The study was a cross-sectional study and the laboratory experiments took place at the Nigerian Institute of Medical Research, Yaba, Lagos, Nigeria.

### Study site

The sampling took place at Amukoko, clinic, Mainland local government area of Lagos State. Amukoko, is an urban slum (informal settlement) geographically located within Longitude 3°23′31.085″ E, Latitude 6°30′9.154″ N; and Longitude 3°22′57.467″ E, Latitude 6°29′28.887″ N, across the 3rd. mainland bridge on the coast of the mainland, Lagos, and is said to harbour about 400,000 humans population [27].

### Study population

The sample size was purposive and time-bound. A total of 232 consecutive pregnant women attending the antenatal clinic at the Amukoko, clinic were recruited. Each participant was unrepeated. The study was conducted from April 2019 to Sept. 2020.

### Criteria

#### Inclusion

All pregnant women in any trimester who consented participated. All those whose symptoms were suggestive of BV were specifically referred by the clinician after examination.

#### Exclusion

All pregnant women who declined consent were excluded. Patients who admitted being on antibiotics 3 weeks before presenting at the clinic were excluded and others with blood spotting and or were diagnosed with Human Immunodeficiency Virus (HIV) infection were excluded.

### Ethical approval

The proposal for the study was reviewed and ethical approval was granted by the Institutional Review Board (IRB), Nigerian Institute of Medical Research (NIMR), Yaba, Lagos, Nigeria on the 13th. April, 2019.

### Clinical staging and sample collection

The clinician conducted the clinical examination on each patient in a private room at the Amukoko clinic, looking for vaginal discharge and its nature (consistency and homogeneity), colour and odour. For each patient, a semi-structured questionnaire was used to gather the socio-demographic, practices/attitudes, and clinical information after thorough explanations by the clinician who performed the examination: specifically highlighting the nature, benefits, and overall aim of the study. It was emphasised that each participant was at liberty to decline participation and that there was no consequence for not participating. Two High Vaginal Swab (HVS) samples each were collected from the patient using a sterile plastic vaginal speculum (QDMH 1012, China) and sterile swab stick (IndiaMART). Briefly: Each sterile swab was saturated with vaginal fluid, by using the speculum to dilate the vaginal orifice, and the sample was taken from the lateral vaginal wall or the posterior fornix.

The samples were labelled and transported at room temperature to the laboratory for processing. One sample was employed for BV and TV microscopy and the second sample for culture.

### Specimens processing

#### Screening for PH

The vaginal fluid-saturated swab was rotated several times on P^H^ colour strip immediately after collection at the clinic’s side laboratory, and instantly change was matched with the colour chart: if the pH level was ≥ 4.5, a plus sign ( +) is recorded indicating a provisional positive case or negative (-) for negative case [28].

#### Wet mount for clue cells and mobile flagellate detection

Wet mount procedure was employed to identify clue cells and TV. Briefly: About 2- 3 mL of newly prepared sterile physiological saline was added into one of the swab containers and gently vortexed. A drop of suspension of the HVS was placed on a clean grease-free glass slide and covered with a cover glass. The preparation was examined microscopically using the 40 × objective lens [29]. Clue cells were identified as epithelial cells from the vagina that appeared fuzzy without sharp edges under the microscope × 40 objective. Clue cells change to this fuzzy look as a result of bacterial presence.

#### Whiff’s test

Whiff’s test is one of the Amstel’s criteria for diagnosis of BV. Briefly: a drop of 10% potassium hydroxide (KOH) was put on a clean glass slide and a swab saturated with vaginal fluid/discharge was rolled on the slide. The preparation was whiffed for the release of a fishy odour indicative of the presence of volatile amines such as trimethylamine and then was recorded as positive or negative, which was confirmed by at least two other laboratory staff.

#### Bacterial vaginosis diagnosis using Amsel’s criteria

According to Amsel’s criteria, clinical diagnosis of BV requires essentially three of the understated four symptoms or signs:

- Homogeneous, thin discharge (milk-like consistency) that smoothly coats the vaginal walls (as was observed by the clinician during sample collection)
- Clue cells harboring adherent bacteria microscopically confirmed.
- pH of vaginal fluid > 4.5
- A positive whiff’s test [9].

#### Examination of the wet preparation for TV

Direct microscopic examination of the wet preparation was employed for the identification of *T. vaginalis*: simple detection of pear-shaped flagellate (trophozoites) with jerky movement according to Cheesbrough [29].

#### High vaginal swab (HVS) culture

Each HVS sample was cultivated on enriched agar (Blood & chocolate) in duplicate, selective agar/indicator (MacConkey) and specific agar (Sabraud Dextrose Agar for isolation of yeast-like cells) at the bacteriology laboratory at NIMR. The plates were incubated both aerobically and in microaerophilic condition (for the duplicate on the enriched media) at 25 ± 2º C for 18 to 24 h. Morphotyping, characterisation and identification of culture isolates were done according to the standard bacteriological techniques [29].

The antibacterial susceptibility profile was conducted using the Kirby Bauer disc diffusion method using Mueller Hinton Agar (MHA) [29]. The antibiotics profiles were interpreted using Clinical Laboratory Standard Institute (CLSI) interpretative criteria [30].

## Results

Two hundred and thirty-two (232) pregnant women, with a mean age of 28.11 ± 7.08 years were screened. The Socio-demographic characteristics of the pregnant women studied are expressed in Table 1. The majority (88%) were of age range 15–35 years, 69% had at least a secondary education and 85% were associated with a kind of trading occupation. About 84.5% of them were in a monogamous relationship and only 15.5% were polygamous. A majority, 163 (70.2%) claimed to have had one sex partner, while 12.1% had two or multiple bed mates. Forty-nine point five (49.5%) were in their third gestation period, 20.7% in their second and 9.5% did not know their exact gestation period (Table 2).

**Table 1:** Socio-demographic characteristics of the pregnant women studied from Amukoko, area of Lagos state

**Characteristics**	**Frequency (per cent)**
**Age group in years**	
15–35	202 (87.1)
36–40	26 (11.2)
> 40	04 (1.7)
Mean age ± SD	28.11 ± 7.08
**Educational Status**	
Primary	52 (22.4)
Secondary	166 (71.6)
Tertiary	14 (6.0)
**Occupation**	
Trading	197 (85.0)
Public servant	12 (5.1)
Student	13 (5.6)
Full time housewife	10 (4.3)
**Marital status**	
Monogamy	196 (84.5)
Polygamy	36 (15.5)
**Number of sex partners**	
One sex partner	163 (70.2)
Two or more partners	28 (12.1)
Declined response	41 (17.7)
**Gestation period**	
First trimester	48 (20.7)
Second trimester	47 (20.3)
Third trimester	115 (49.5)
Undeclared	22 (9.5)

Key: *SD* Standard Deviation

**Table 2:** Age distribution and pattern of microbial infections of pregnant women studied

**Age Range in Years**	**No (%)**	**No (%)** **Infected with bacterial organism**	**No (%)** **Infected with TV**	**No (%)** **Bacterial &TV co-infection**
15–35	202 (87)	33 (14.22)	36(15.5)	12 (5.2)
36–40	26 (11.2)	0	0	0
> 40	4 (1.7)	0	0	0
**Total**	**232 (100)**	**33(14.22)**	**36 (15.5)**	**12 (5.2)**

Key: *TV Trichomonas vaginalis*

Out of the 232 subjects studied, 81 (34.9%) had microbial organisms isolated or seen from their specimens. Only 19 (8.2%) of such were classified as having bacterial vaginosis, applying Amsel’s interpretative criteria, and were mainly from the active reproductive age group 15–35 years of age; while those within the age range of > 40 years contributed none (Table 2).

Of the 81 infected ones, 33 (40.8%) had only bacterial infection, 36 (44.4%) had TV alone and 12 (14.8%) were those that had bacterial co-infected with TV. Table 3 shows the association of socio-demographic characteristics and other factors with the prevalence of BV and co-infection with flagellate among the pregnant women studied. From the clinical records, the population that was classified as having UTI or vaginitis was only 46 (20.7%) and had symptoms related to the same as a result of the presence of a bacterial pathogen, vulvovaginal candidiasis, flagellate, or both.

**Table 3:** Association of socio-demographic characteristics and other factors with prevalence of BV and co-infection with flagellate among the pregnant women studied

**Variables**	**No Positive (%)**	**No Negative (%)**	**Total No (*N* = 232)**	**X**^**2**^	***P*-value**
	**Bacterial Vaginosis & TV co-infection**				
**Age groups(years)**					
15–35	81(100)	121(80.1)	202	7.9	0.005*
36–40	0(0)	26(17.2)	26		
> 40	0(0	4(2.6)	4		
**Educational Status**					
Primary	21(25.9)	31(20.5)	52	1.92	0.166
Secondary	60(74.1)	106(70.2)	166	12.75	0.0001
Tertiary	0(0)	14(9.3)	14		
**Occupation**					
Trading	78(96.3)	119(78.8)	197	8.533	0.003*
Public servant	3(3.7)	9(6.0)	12	3	0.083
Student	0(0)	13(8.6)	13		
Full time housewife	0(0)	10(6.6)	10		
**Marital status**					
Monogamy	76(93.8)	120(79.5)	196	9.878	0.002*
Polygamy	5(6.2)	31(20.5)	36	18.778	0.0001*
**Number of sex partners**					
One sex partner	61(75.3)	102(67.5)	163	10.313	0.001*
Two or more sex partners	0(0)	28(18.5)	28		
Declined response	20(24.7)	21(13.9)	41	0.024	0.876
**Gestation period**					
First trimester	24(29.6)	24(15.9)	48	0.0001	0.98
Second trimester	16(19.8)	31(20.5)	47	4.787	0.029*
Third trimester	41(50.6)	74(49.0)	115	9.47	0.002*
Undeclared	0(0)	22(14.6)	22		

Key: *Statistically significant; X^2^ = Chi-square, Percent (%) based on the total number of positive or negative microbial detections

A majority (74.1%) of those infected had at least a secondary education. Only 29.1% had primary education. The study observed age (15–35 years) related association between vaginosis/ TV co-infection (X^2^ = 7.9; *P* = 0.005) (Table 3). Pregnant women with symptoms of vaginitis or UTI, bacterial vaginosis/co-infection with flagellate were significantly associated with female traders (X^2^ = 8.5; *P* = 0.003) and were more associated with those from polygamous relationships than those in a monogamous relationship (X^2^ = 18.79, *P* = 0.0001), (Table 2). Pregnant women in their 3^rd^ and 2^nd^ trimester were more significantly associated with vaginal infection (X^2^ = 9.47, *P* = 0.002; X^2^ = 4.79, *P* = 0.029) respectively. There were no microbial isolates or parasites seen among the pregnant women that claimed to have had multiple sex partners and 24.7% that did not declare the number of sex partners were not significantly associated with vaginal infection (X^2^ = 0.024; *P* = 0.876).

On the distribution of the gestation period and microbial infections, most pregnant women in their 3^rd^ trimester were infected with the flagellate and more of those in their 1^st^, trimester were infected with bacteria, and by extension the population with the most bacterial vaginosis The highest bacterial & TV co-infection was among pregnant women in their last trimester.

**Fig. 1:**
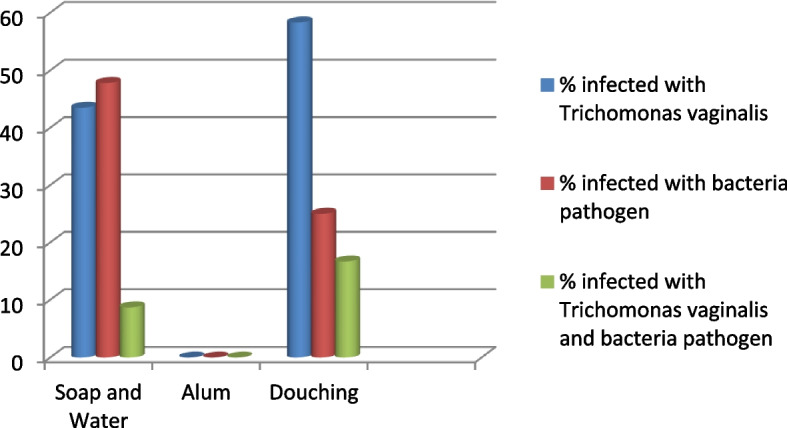
The pattern of microbial infection among the participants that applied different methods for vaginal hygiene

The outcome of the evaluation of vaginal hygiene practices and the microbial ecology of the study population are expressed in Fig. 1.

Out of the 81 participants with microbial infection 69 (85.2%) volunteered information on the use of soap and water for vaginal hygiene. Many (43.5%) of those with TV infection, 47.8% of those with bacterial infection and 8.7% of the population with bacterial and TV co-infection admitted cleaning their vagina with soap and water respectively. Only a few (14.8%) claimed to have indulged in douching. No participant admitted employing alum for the same purpose.

A total of 34 bacterial organisms were isolated from the participants (Table 3).

Pseudomonas spp. (10) and *E. coli* (9) had the highest occurrence, Table 4. However, one patient had *E. coli* and Proteus spp. bacterial co-infection. Also, 3 other pregnant women studied had bacteria and candida spp. co-infection, and only one participant had multiple infection of a flagellate, bacterium and candida species.

**Table 4:** The occurrence of bacterial organisms (*N* = 34)

Organism (code)	Occurrence
Pseudomonas spp. (P)	10
*Escherichia coli* (E)	09^b^
*Streptococcus pyogenes* (STR)	06
Bacteriods (BAC)	03^a^
Gardnerella (GAR)	02^b^
Enterobacta (ENT)	02^b^
Proteus spp. (PRO)	02^a^

Key:

^a^Bacterial multiple isolates from a patient

^b^Bacterial co-infection with Fungi (Candida species)

The antibiogram of some of the apparently significant bacterial isolates are as presented in Tables 5 and 6.

**Table 5:** Antibiotic susceptibility pattern of the Gram negative bacterial isolates, using CLSI interpretative criteria [30]

**Organism**	**Antibiotics and zones of inhibition in mm (interpretation)**							
NO	CXM 30 µg	CFM 30 µg	Amox/Clav. 30 µg	TM 30 µg	CIP 5 µg	CAZ 30 µg	IMP 10 µg	GEN 10 µg
P1	30 (S)	16 (S)	20 (S)	0 (R)	25 (S)	0 (R)	0 (R)	10 (R)
P2	26 (S)	18 (S)	11 (R)	14 (R)	28 (S)	13 (R)	9 (R)	16 (S)
P3	10 (R)	14 (S)	14 (I)	13 (R)	26 (S)	16 (R)	22 (I)	13 (I)
P4	28 (S)	12 (I)	10 (R)	15 (S)	16 (I)	0 (R)	18 (R)	11 (R)
P5	22 (I)	11 (!)	16 (I)	0 (R)	24 (S)	5 (R)	20 (I)	10 (R)
P6	30 (S)	9 (R)	0 (R)	16 (S)	25 (S)	20 (I)	33 (S)	14 (I)
P7	24 (I)	0 (R)	22 (S)	17 (S)	14 (R)	0 (R)	30 (S)	10 (R)
P8	30 (S)	15 (I)	0 (R)	12 (R)	23 (S)	19 (I)	0 (R)	19 (S)
P9	17 (R)	22 (S)	14 (I)	10 (R)	19 (I)	0 (R)	22 (I)	11 (R)
P10	30 (S)	17 (S)	0 (R)	12 (R)	22 (S)	15 (R)	18 (R)	11 (R)
E1	33 (S)	20 (S)	22 (S)	29 (S)	40 (S)	30 (S)	24 (S)	18 (S)
E2	38 (S)	22 (S)	20 (S)	30 (S)	34 (S)	35 (S)	18 (R)	8 (R)
E3	12 (R)	21 (S)	17 (I)	13 (I)	20 (I)	20 (I)	40 (S)	12 (R)
E4	13 (R)	14 (S)	14 (I)	13 (R)	25 (S)	16 (R)	25 (S)	13 (I)
E5	12 (R)	12 (I)	11 (R)	15 (S)	16 (I)	0 (R)	18 (R)	11 (R)
E6	22 (I)	14 (S)	16 (I)	9 (R)	24 (S)	15 (R)	20 (I)	20 (S)
E7	32 (S)	23 (S)	22 (S)	29 (S)	40 (S)	30 (S)	24 (S)	18 (S)
E8	36 (S)	21 (S)	20 (S)	30 (S)	35 (S)	37 (S)	18 (R)	8 (R)
E9	13 (R)	20 (S)	17 (I)	13 (I)	20 (I)	20 (I)	30 (S)	13 (I)
ENT1	10 (R)	14 (S)	14 (I)	13 (R)	21 (I)	16 (R)	22 (I)	13 (I)
ENT2	23 (I)	12 (I)	17 (S)	15 (S)	16 (I)	0 (R)	18 (S)	11 (R)
PRO1	22 (I)	11 (I)	16 (I)	11 (R)	24 (S)	15 (R)	22 (I)	19 (S)
PRO2	30 (S)	20 (S)	0 (R)	12 (R)	33 (S)	16 (R)	20 (I)	20 (S)
BAC1	Routine antibogram not indicated							
BAC2								
BAC3								
GAR								

Key: *CXM* Cefuroxime (Second-generation cephalosporin), *CFM* Cefepime (fourth-generation cephalosporin), *Amox/clav* Amoxicillin/ Clavulanic acid (Penicillin and beta-lactamase inhibitors), *TM* Tobramycin (Aminoglycoside), *CIP* Ciprofloxacin (Fluoroquinolone), *CAZ* Ceftaxidime (Cephalosporin), *IMP* Imipenem (Carbapenem), *GEN* Gentamicin (Aminoglycosides), *CLIS* Clinical Laboratory Institute Standard

**Table 6:** Antibiotic susceptibility pattern of the Gram positive bacterial isolates

**Organism**	**Antibiotics and zones of inhibition in mm (interpretation)**							
**NO**	**CTR**	**ERY**	**CTX**	**OFX**	**Amox/Clav**	**CAZ**	**CRX**	**GEN**
STR1	22 ( I)	35 (S)	12 (R)	22 (S)	30 (S)	0 (R)	30 (S)	28 (S)
STR2	28 ( S)	32 (S)	27 (S)	10 (R)	34 (S)	18 (I)	27 (S)	33 (S)
STR3	32 ( S)	29 (S)	13 (R)	14 (R)	38 (S)	12 (R)	26 (S)	15 (S)
STR4	29 ( S)	34 (S)	30 (S)	15(R)	36 (S)	0 (R)	33 (S)	30 (S)
STR5	33 ( S)	30 (S)	12 (R)	28 (S)	26 (S)	23 (S)	28 (S)	13 (I)
STR6	40 ( S)	33 (S)	11 (R)	10 (R)	38 (S)	15 (R)	37 (S)	14 (I)

Key: *CAZ* Ceftaxidime (Cephalosporin), *GEN* Gentamicin (Aminoglycosides), *CTR* Ceftriaxone (third generation cephalosporin), *ERY* Erythromycin (Macrolide), *CTX* Cefotaxime (Cephalosporin), *OFX* Ofloxacin (fluoroquinolone), *CRX* Cefuroxime (Cephalosporin)

The streptococcus isolated from the pregnant women showed 100% susceptibility to 3 antibiotics tested (ERY, AMOX/CLAV & CRX), 90% to CTX and 67%. The strains demonstrated 67% in vitro resistance to only OFX and CAZ (Table 6).

**Fig. 2:**
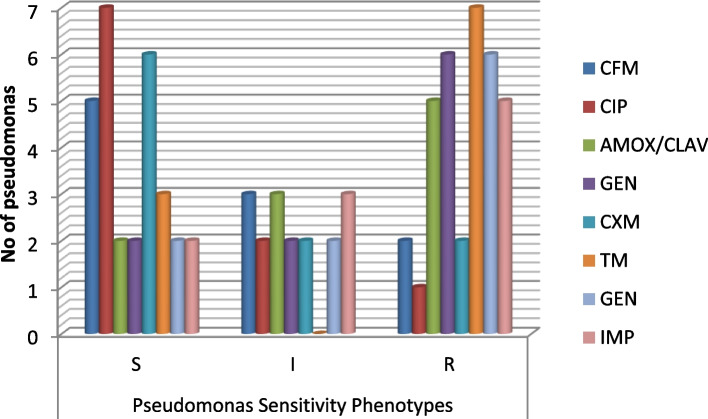
Pseudomonas sensitivity & resistance phenotypes (*n* = 10). Key: R = Resistant, I = intermediate, S = Sensitive

Some of the pseudomonas strains are merely sensitive to CIP and CXM among others, while many are among intermediate dose dependent interpretative category and almost all are resistant to TM (Fig. 2).

**Fig. 3:**
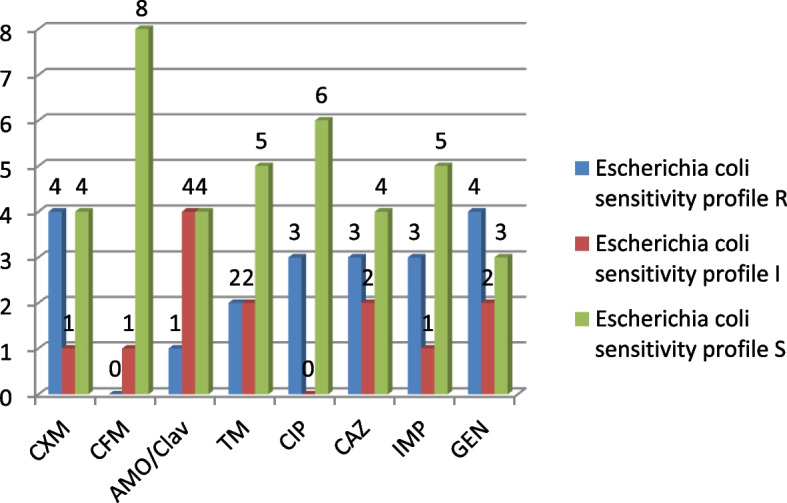
*Escherichia coli* sensitivity & resistance phenotypes (*n* = 9)

Eight out of 9 of the isolates were sensitive to Cefepime and 4 were resistant to gentamycin (Fig. 3).

**Fig. 4:**
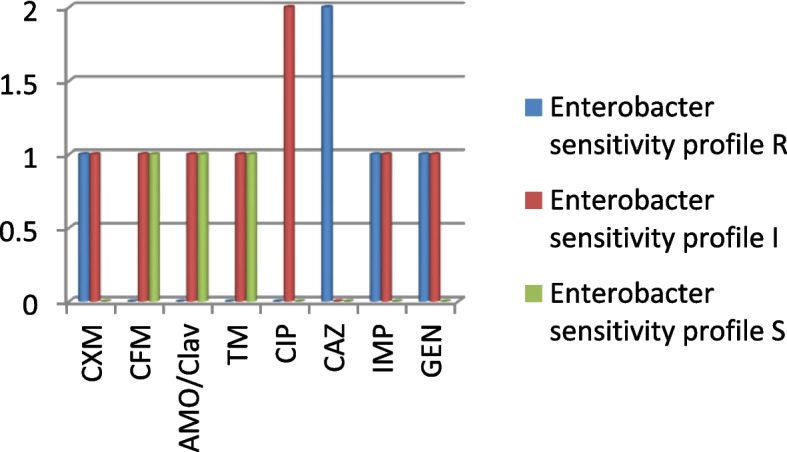
Sensitivity profile of Enterobacter species (*n* = 2)

Both Enterbacter strain are resistant to CAZ and intermediate to CIP, while one was sensitive to CFM, AMOX/CLAV and TM (Fig. 4).

**Fig. 5:**
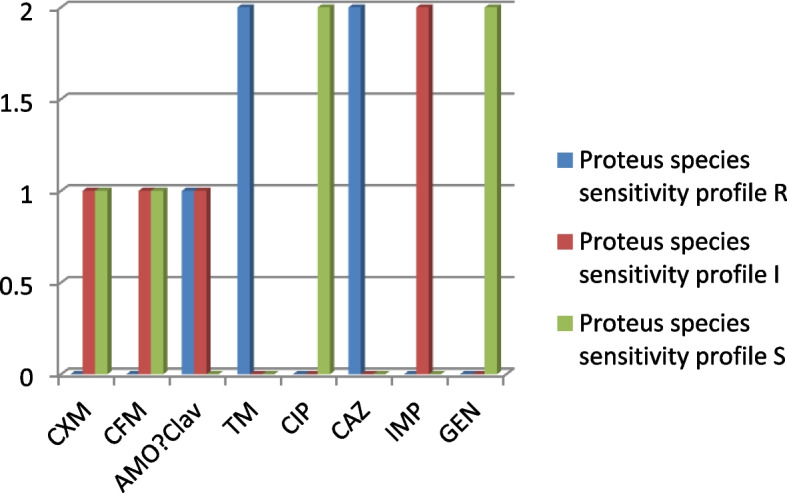
Proteus species antibiogram (*n* = 2)

The Proteus isolates are both sensitive to CIP and GEN, and are resistant to TM and CAZ (Fig. 5).

## Discussions

In this study, we review some often misrepresented terms such as bacterial vaginosis which is the alteration of the vaginal bacterial ecosystem or microbiota, and vaginitis simply put, is the inflammation of the vagina that can result in pain, itching, and discharge; which may be occasioned by reproductive carnal infections (e.g. gonorrhoea, syphilis, candidiasis or trichomoniasis). These conditions may be symptomatic or asymptomatic, and are more common among women of reproductive age, with variable symptoms like vaginal discharge, itching, irritation, spiteful odour and discomfort [24, 31]). In this study the prevalence of bacterial vaginosis infection was high among the ages between 15–35 years, mean age 28.11 ± 7.08. The report is in agreement with the global women reproductive age of 15–49 years [32]. These are within the range of other reports: Amadi et al. [33], reported 21–30 years, and Udogu et al. [33] reported 27.25 ± 6.09 years in Nigeria, However, the prevalence of BV varies: the 8.2% in this report is far lower than Amadi’s prevalence rate of 20.6% in 2013 and Udogu’s prevalence rate of 21.7% in the South Eastern part of Nigeria in 2022. Konadu et al. [34] reported a BV prevalence of 30.9% and 56.4% of ‘at least one vaginal contaminant’ in Ghana. In America, variable prevalence of BV & TV was reported ranging from 60 to 80% [35]. On a general note, our study found actual microbial contamination of 34.9%: 14.22% were specific for bacterial organisms, 15.5% were infected by TV alone and only 5.2% were bacteria co-infected with TV. This report appears to agree with the report of Sena et al. [35] in 2018 of 15.6% TV prevalence among American sexually active females aged 15–24 years of age. However, this report of 15.5% presence of TV is higher than the 1.4% reported by Konadu et al. [34] in Ghana. In America, variable prevalence of BV & TV was reported ranging from 60 to 80% [35]. Generally, in Africa, Asia, and Latin America, the prevalence of > 20%, 11%, and 12–24.2% of TV were respectively reported among women [24]. Essentially, the discrepancies may be associated with variability in study design, attitude, and sanitary activities among different populations. Again, the difference in the diagnostic methods may be a major factor of variability [35–38]. For instance, Barbosa et al. [38] reported that wet mount had lower sensitivity when compared with PCR method for the diagnosis of flagellate. Barbosa et al. [38] further reported TV prevalence of 27.8% and 7.41% for PCR and wet mount respectively among Brazilian women.

Therefore the low prevalence report of bacterial and flagellate co-infection could be a result of under diagnosis.

The study of co-infection is important since different pathogens have been reported to interact within the host microbiota with deleterious or symbiotic effects on other pathogens or the host [12]. For instance, it was reported that TV and BV organisms can reliably cohabit with the female vagina; both profiting from the increased vaginal P^H^ and anaerobic environment created by the extinction of vaginal normal flora [13]. Specifically, the parasite was reported to mediate adherence to epithelial cells to colonize the human host, interface with the host immune system and vaginal microbiota, causes host tissue damage and alter the vagina ecology [39]. However, according to Governder et al. [40] the role of bacterial–protozoan and trichomonas-virus symbiosis remains unclear. Therefore, syndemism and comorbidity of untreated BV co-infected with other pathogens may progress to vaginitis or urethritis according to Mirmonsef et al. [14]; this further underscores the need for this study.

From our report, the majority (50.6%) of pregnant women infected by microbes were in their third gestation period. On the risk factors of BV and TV co-infection, our report showed a statistically significant association of age group 15–35 years of age with a higher prevalence of BV and TV co-infection (X^2^ = 7.9; *P* = 0.005) Table 3. Analysis of independent factors like educational status, occupation, and multiple sex partners was significantly tilted towards those with intermediate educational status (X^2^ = 12.75; *P* = 0.0001), itinerant traders (X2 = 8.533; *P* = 0.003) and more with those in a polygamous relationship (X2 = 18.78; *P* = 0.0001). These are similar to the report of Udeogu et al. [33] from south eastern region of Nigeria. Although their report associated BV more with pregnant women who had primary education and those without employment, this report associated BV with those who had secondary education and were itinerant hawkers. The differences may be attributed to variations in educational and employment opportunities in Nnewi (a commercial city) versus Amukoko, (an urban slum). Also, Konadu et al. [34] in a univariate analysis of their study reported marital, educational and occupational characteristics as independent variables associated with BV, this is in agreement with our findings. However, the age group of their study population included the age range > 35 years of age as a risk factor for BV and that varies with our report of 15–35 years of age, The variability may be a result of study design and study setting: the Konadu’s study was in a rural setting while the present was designed for urban slum. The age variability when compared with the study by Udeogu et al. [33] varies age-wise as well, and this may be a result of differences in the patients’ recruitment, while this study recruited clients on routine antenatal attendance, Udeogu et al. [33] recruited pregnant women with symptoms of vaginitis attending gynaecological clinic. Furthermore, this study reports an association between BV and TV co-infection with women in their second and third gestation periods (X^2^ = 4.79; *P* = 0.0029) and (X^2^ = 9.97; *P* = 0. 002 respectively as against ‘no association’ reported by Konadu et al. [34] despite concomitance in the study design. It is pertinent to note that this study did not recover bacteria or flagellate from pregnant women 36 ≥  40 years of age contrary to 18 (20.2%) reported by Konadu et al. [34] in Ghana and 1 (16.7%) by Udeogu et al. [33] in Nigeria. The most probable reason may be the geographical setting as this study tends to agree with the report of Udeogu et al. [33] in Nigeria. Conversely, it could be inferred that elderly women with more pregnancy tantrum experiences are more likely to pay attention to good hygiene and proper antenatal care as against younger ones who are more rapacious with sex enterprise as suggested by Udeogu et al. [33] and Muzny et al. [41].

Curiously, the study did not record any vaginal microbial contamination from the population that claimed to have had multiple sex bed mates. However, the study reports 24.7% microbial contamination of pregnant women who withheld information on multiple sex partners. This is at variance with the report of Huang et al. [42] who associated multiple sex partners with BV.

Etiologically, this study reports core BV organisms isolated as Bacteriods and Gardnrella species, these are consistent with the report of many scholars [4, 5, 42]. However, the exact cause of BV remains uncertain, because several hypothetical prototypes have been published, including *G. vaginalis*, *P. bivia*, *A. vaginae*, and Megasphaera species. Others are Prevotella spp., *Atopobium vaginae*, and Sneathia spp. [5, 43]. Also, *Chlamydia trachomatis* and genital mycoplasmas were associated with BV [28, 33]. Contrastingly some epidemiological data thought that BV should be regarded as STI [44–46]. However, the Center for Disease Control of America, and some other health authorities do not support routine screening for BV in asymptomatic pregnant women, suggesting that the condition is not a serious health issue [4, 47]. Although, treatment is indicated in symptomatic patients to gain relief from the vaginal symptoms and reduction in the risk of acquiring co-infection with either *C. trachomatis*, *N. gonorrhoeae*, *T. vaginalis*, *M. genitalium,* or viruses [9, 48].

According to the CDC [9], the standard treatment for BV is antibiotics such as metronidazole or tinidazole and clindamycin in symptomatic clients and these drugs are said to be efficacious, although some resistant strains have been reported in some cases [5].

In addition to BV agents, this report associates some pregnant women from the Amukoko, slum with Pseudomonas spp., *E. coli*, Proteus spp. Enterobacter, and Gram-positive *Streptococcus pyrogens* infections. These organisms have been implicated with STI or PID in pregnant women [44–46]. Recovering Pseudomonas as the most prevalent pathogen is not surprising as the study environment is surrounded by almost stagnant water and Pseudomonas is known to be found in water, including lakes and stagnant water [49, 50]. Routine antibiograms are recommended for these organisms and the Pseudomonas showed susceptibility to CIP (7) and CXM (6) out of ten isolates (Fig. 2 and Table 5). This is in agreement with previous reports that ceftazidime or ciprofloxacin is effective against Pseudomonas species associated with UTI [51]. While ceftriaxone, clindamycin, erythromycin and azithromycin are indicated for *E. coli* isolates from UTI, this study reports cefepime, ciprofloxacin and imipenem as the most effective (Fig. 3). Our study did not include clindamycin and azithromycin since they are not popular or available in antimicrobial stewardship within the study area. In all, relatively high resistance exhibited by the isolates including the Proteus (Fig. 5) and Enterobacter species (Fig. 4) is not surprising since Bostwick et al. [52] reported in 2016 that antimicrobial resistance genes were identified in all drug classes tested: macrolides, lincosamides, aminoglycosides among others and further revealed a fourfold-higher frequency of AMR genes among bacterial isolates from women with BV when compared with those without BV [52]. The Gram-positive streptococcus strains showed sensitivity toward CRX, ERY, CTR, and AMOX/CLAV (Table 6). Conversely, Raabe and Andi [53] reported that streptococcus isolated from UTI is generally susceptible to Penicillin G and other beta-lactam antibiotics, including ampicillin, 1st, 2nd and 3rd. first-generation cephalosporins, and carbapenems. However, this does not foreclose variations in the level of activity among the classes of antibiotics. The differences may not be unconnected with peculiarities in antibacterial selective pressure among communities, the rapid spread of resistomes and the dynamism of mobile genetic elements within the microbiome.

## Conclusion and recommendation

There is a relatively low prevalence of BV and flagellate co-infection in the community studied; our report may not be unconnected with socio-economic and educational facilities improvement in Lagos State. Recall that for almost a decade, the Lagos State government implemented a free educational policy and has carried out a good public health campaign against maternal mortality. These policies presuppose improved health awareness of good antenatal care. This study advocates for the sustainability of all socio-economic and primary health improvement programme of Lagos State and implore other state and Local government authority to emulate the same. There is a relatively low prevalence of BV and flagellate co-infection in the community studied.

### Recommendation

We recommend screening of antenatal women with underlying symptoms for BV and flagellates co-infection to avoid its progression to vaginitis. Trichomoniasis on the other hand is said to be associated with transmissible urogenital infections such as viruses e.g. human papillomavirus (HPV), bacteria e.g. Neisseria organism and other microbes. From other peer reports the overall consequences of risk of untreated TV infection include the risk for preterm delivery, low birth weight of new born, premature rupture of membranes and that the pregnant women are prone to developing pelvic inflammatory disease (PID).

### Study limitations

The study design has some limitations as the study excluded pregnant women who are not on routine antenatal care or those who attended private clinics and in particular, the conservative group that still believes and patronizes local health practitioners and traditional birth attendants. Again, the BV test method is a source of limitations, since the PCR method has a more significant case recovery tendency when compared with the wet mount employed. These certainly will impede generalisation of this report. We therefore, recommend a funded generalizable study, particularly, to decipher the effects of bacterial co-infection with flagellates on pregnancy complications.

### Supplementary Information

Supplementary Material 1.

## Data Availability

All data generated and analysed during this study are included in the published article. A supplementary file containing the raw data of the socio-demographic characteristics and other factors influencing BV and BV co-infection with TV; including the microbial/ flagellate occurrence among the pregnant women studied is attached.

## Abbreviations

BV: Bacterial Vaginosis
TV: Trichomonas Vaginalis
CDC: Center for Disease Control
HVS: High Vaginal Swab
CLSI: Clinical Laboratory Institute’s Standard
HPV: Human Papilloma Virus
PID: Pelvic Inflammatory Disease
IRB: Institutional Review Board

## References

[1] Lin P, et al. Vaginal PH value for clinical diagnosis and treatment of common vaginitis. Diagnostics. 2021;11(11):1996. 10.3390/diagnostics1111199. 34829343 10.3390/diagnostics1111199 PMC8618584

[2] Hillier S, Holmes K, et al. Bacterial vaginosis. In: Holmes K, Sparling P, Mardh P, et al., editors. Sexually transmitted diseases. 3rd ed. New York: McGraw-Hill; 1999. p. 563–86.

[3] Centers for Disease Control and Prevention. Trichomoniasis – CDC fact sheet. 2017. https://www.cdc.gov/std/trichomonas/STDFact-Trichomoniasis.htm.

[4] USPSTF (US Preventive Services Task Force). Screening for bacterial vaginosis in pregnant persons to prevent preterm delivery: US Preventive Services Task Force recommendation statement. JAMA. 2020;323(13):1286–92. 10.1001/jama.2020.2684. 32259236 10.1001/jama.2020.2684

[5] Muzny CA, Sobel JD. The role of antimicrobial resistance in refractory and recurrent bacterial vaginosis and current recommendations for treatment. Antibiotics. 2022;11:500. 10.3390/antibiotics11040500. 35453251 10.3390/antibiotics11040500 PMC9024683

[6] Workowski KA, Bachmann LH, Chang PA, et al. Sexually transmitted infections treatment guidelines. MMWR Recomm Rep. 2021;70(4):1–187. 34292926 10.15585/mmwr.rr7004a1 PMC8344968

[7] Centers for Disease Control and Prevention. STDs & pregnancy—CDC fact sheet. 2011. Retrieved March 28, 2012, from https://www.cdc.gov/std/pregnancy/STDFact-Pregnancy.htm.

[8] AbouChacra L, Fenollar F, Diop K. Bacterial vaginosis: what do we currently know? Front Cell Infect Microbiol. 2022;11:672429. 10.3389/fcimb.2021.672429. Published 2022 Jan 18. 35118003 10.3389/fcimb.2021.672429 PMC8805710

[9] CDC Facts & Brochures, 2021 The Facts - Trichomoniasis (cdc.gov).

[10] McGregor JA, French JI. Bacterial vaginosis in pregnancy. Obstet Gynecol Surv. 2000;55(5 Suppl 1):S1-19. 10.1097/00006254-200005001-00001. PMID: 10804540. 10804540 10.1097/00006254-200005001-00001

[11] Schumann JA, Plasner S. Trichomoniasis. [Updated 2023 Jun 12]. In: StatPearls. Treasure Island: StatPearls Publishing; 2023. Available from: https://www.ncbi.nlm.nih.gov/books/NBK534826/.

[12] Griffiths EC, Pedersen AB, et al. The nature and consequences of co-infection in humans. J Infect. 2011;63(3):200–6. 10.1016/j.jinf.2011.06.005. Accessed 19 Mar 2023. 21704071 10.1016/j.jinf.2011.06.005 PMC3430964

[13] Loveless M, Myint O. Vulvovaginitis- presentation of more common problems in pediatric and adolescent gynecology. Best Pract Res Clin Obstet Gynaecol. 2018;48:14–27. 10.1016/j.bpobgyn.2017.08.014. 28927766 10.1016/j.bpobgyn.2017.08.014

[14] Mirmonsef P, et al. The role of bacterial vaginosis and trichomonas in HIV transmission across the female genital tract. Curr HIV Res. 2012;10(3):202. 10.2174/157016212800618165. Accessed 23 Nov 2023. 22384839 10.2174/157016212800618165 PMC3788616

[15] Threstha S. Ecology and evolution of host-pathogen interactions in nature. Am Nat. 2011;164(S5):S1–5. https://www.jstor.org/stable/10.1086/424611.

[16] Knezevic A, Stepanovic S, Cypic M, Jevtovic D, Ranin J, Jovanovic T. Reduced quantity and hydrogen-peroxide production of vaginal lactobacilli in HIV positive women. Biomed Pharmacother. 2005;59(9):521–3. 16271844 10.1016/j.biopha.2005.06.010

[17] Kalia N, Singh J, Kaur M. Microbiota in vaginal health and pathogenesis of recurrent vulvovaginal infections: a critical review. Ann Clin Microbiol Antimicrob. 2020;19:5. 10.1186/s12941-020-0347. 31992328 10.1186/s12941-020-0347 PMC6986042

[18] Fooladi AAI, Khani S, Hosseini HM, et al. Impact of altered early infant gut microbiota following breastfeeding and delivery mode on allergic diseases. Inflamm Allergy Drug Targets. 2013;12(6):410–8. 24304331 10.2174/1871528112666131205113129

[19] Africander D, Louw R, Verhoog N, et al. Differential regulation of endogenous pro-inflammatory cytokine genes by medroxyprogesterone acetate and norethisterone acetate in cell lines of the female genital tract. Contraception. 2011;84(4):423–35. 21920200 10.1016/j.contraception.2011.06.006

[20] Hillebrand L, Harmanli OH, Whiteman V, Khandelwal M. Urinary tract infections in pregnant women with bacterial vaginosis. Am J Obstet Gynecol. 2002;186(5):916–7. 10.1067/mob.2002.123987. PMID: 12015512. 12015512 10.1067/mob.2002.123987

[21] Donders GG, Van Calsteren K, Bellen G, Reybrouck R, Vanden Bosch T, Riphagen I, et al. Predictive value for pretermbirth of abnormal vaginal flora, bacterial vaginosis and aerobic vaginitis during the first trimester of pregnancy. BJOG. 2009;116(10):1315–24. 19538417 10.1111/j.1471-0528.2009.02237.x

[22] World Health Organistion. Global prevalence of incidence of selected curable sexually transmitted infections. 2001. (who.int). 10023347

[23] Bowden FJ, Garnet GP. Trichomonas vaguinalis epidemiology parametising and analyzing a model of treatment intervention. Sex Transm Infect. 2000;76:248–56. 11026878 10.1136/sti.76.4.248 PMC1744187

[24] Peebles K, Velloza J, Balkus JE, McClelland RS, Barnabas RV. High global burden and costs of bacterial vaginosis: a systematic review and meta-analysis. Sex Transm Dis. 2019;46(5):304–11. 10.1097/OLQ.0000000000000972. 30624309 10.1097/OLQ.0000000000000972

[25] Bhakta V, Aslam S, Aljaghwani A. Bacterial vaginosis in pregnancy: prevalence and outcomes in a tertiary care hospital. Afr J Reprod Health. 2021;25(1):49–55. 10.29063/ajrh2021/v25i1.6. 34077110 10.29063/ajrh2021/v25i1.6

[26] Afolabi BB, Moses EO, Oduyebo OO. Bacterial vaginosis and pregnancy outcome in Lagos, Nigeria. Open Forum Infect Dis. 2016;3(1):30. 10.1093/ofid/ofw030 PMC4794946 26989754

[27] Oyinloye MA, Olamiju IO, Popoola OO. Urban renewal strategies in developing nations: a focus on Amukoko, Lagos State, Nigeria. J Geogr Reg Plann. 2017;10(8):229–41. 10.5897/JGRP2017.0631

[28] Charonis G, Larsson PG. Use of pH/whiff test or QuickVue advanced pH and amines test for the diagnosis of bacterial vaginosis and prevention of postabortion pelvic inflammatory disease. Acta Obstet Gynecol Scand. 2006;85(7):837–43. 10.1080/00016340600589776. 16817083 10.1080/00016340600589776

[29] Cheesbrough M. District laboratory practice in tropical countries part 1. 2nd ed. Cambridge: Cambridge University Press; 2009. p. 207–66.

[30] Clinical Laboratory Standard Institute (CLSI). M100: performance standards for antimicrobial susceptibility testing, 27th edition. clsi.org; 2017, WEB500 1ST AVE. PITTSBURGH, PA 15219, USA.

[31] Sobel JD. Bacterial vaginosis. Annu Rev Med. 2000;51:349–55. 10774469 10.1146/annurev.med.51.1.349

[32] WHO. Women of reproductive age (15-49 years) population (thousands). 2023. https://www.who.int/data/gho/indicator-metadata-registry/imr-details/women-of-reproductive-age-(15-49-years).

[33] Udeogu, et al. Prevalence of bacterial vaginosis in pregnant women attending Nnamdi Azikiwe University Teaching Hospital, Nnewi, Nigeria using the complete Amsel’s diagnostic criteria. Afr J Clin Exper Microbiol. 2022;23(3):311–7. 10.4314/ajcem.v23i3.10

[34] Konadu et al. BMC Pregnancy Childbirth. 2019;19:341. 10.1186/s12884-019-2488-z. 10.1186/s12884-019-2488-z PMC6757405 31547803

[35] Sena AC, et al. Bacterial vaginosis and its association with incident *Trichomonas vaginalis* infections: a systematic review and meta-analysis. Sex Transm Dis. 2021;48(12):e192. 10.1097/OLQ.0000000000001537. Accessed 16 Oct 2023. 34433796 10.1097/OLQ.0000000000001537 PMC8594503

[36] Roth AM, Williams JA, Ly R, et al. Changing sexually transmitted infection screening protocol will result in improved case finding for *Trichomonas vaginalis* among high-risk female populations. Sex Transm Dis. 2011;38:398–400. 21217417 10.1097/OLQ.0b013e318203e3ce

[37] Squire DS, et al. *Trichomonas vaginalis* infection in southern Ghana: clinical signs associated with the infection. Trans R Soc Trop Med Hyg. 2019;113(7):359–69. 30989196 10.1093/trstmh/trz019

[38] Barbosa S, et al. Prevalence and factors associated with Trichomonas vaginalis infection in indigenous Brazilian women. PLoS One. 2020;15(10):e0240323. 10.1371/journal.pone.0240323. Accessed 16 Oct 2023. 33064733 10.1371/journal.pone.0240323 PMC7567381

[39] Mercer F, Johnson PJ. Trichomonas vaginalis: pathogenesis, symbiont interactions, and host cell immune responses. Trends Parasitol. 2018;34(8):683–93. 10.1016/j.pt.2018.05.006. Accessed 24 Mar 2023. 30056833 10.1016/j.pt.2018.05.006 PMC11132421

[40] Govender Y, Chan T, Yamamoto HS, Budnik B, Fichorova RN. The role of small extracellular vesicles in viral-protozoan symbiosis: lessons from Trichomonasvirus in an isogenic host parasite model. Front Cell Infect Microbiol. 2020;10:591172. 10.3389/fcimb.2020.591172. Published 2020 Nov 5. 33224901 10.3389/fcimb.2020.591172 PMC7674494

[41] Muzny CA, Sunesara IR, Martin DH, Mena LA. Sexually transmitted infections and risk behaviors among African American women who have sex with women: does sex with men make a difference? Sex Transm Dis. 2011;38(12):1118–25. 10.1097/OLQ.0b013e31822e6179. 22082722 10.1097/OLQ.0b013e31822e6179

[42] Huang Y, et al. Multiple sexual partners and vaginal microecological disorder are associated with HPV infection and cervical carcinoma development. Oncol Lett. 2020;20(2):1915–21. 10.3892/ol.2020.11738. Accessed 19 Oct 2023. 32724435 10.3892/ol.2020.11738 PMC7377087

[43] Muzny CA, Taylor CM, Swords WE, Tamhane A, Chattopadhyay D, Cerca N, Schwebke JR. An updated conceptual model on the pathogenesis of bacterial vaginosis. J Infect Dis. 2019;220:1399–405. 31369673 10.1093/infdis/jiz342 PMC6761952

[44] Swidsinski A, Doerffel Y, Loening-Baucke V, Swidsinski S, Verstraelen H, Vaneechoutte M, Lemm V, Schilling J, Mendling W. Gardnerella biofilm involves females and males and is transmitted sexually. Gynecol Obstet Investig. 2010;70:256–63. 21051845 10.1159/000314015

[45] Muzny CA, Schwebke JR. Gardnerella vaginalis: still a prime suspect in the pathogenesis of bacterial vaginosis. Curr Infect Dis Rep. 2013;15:130–5. 23371405 10.1007/s11908-013-0318-4

[46] Onoh R, Umeora O, Egwuatu V, Ezeonu P, Onoh T. Antibiotic sensitivity pattern of uropathogens from pregnant women with urinary tract infection in Abakaliki, Nigeria. Infect Drug Resist. 2013;6:225–33. 10.2147/IDR.S46002. Published 2013 Dec 2. 24324344 10.2147/IDR.S46002 PMC3854917

[47] Center for Disease Control (CDC). Sexually transmitted infections treatment guideline. 2021. https://www.cdc.gov/std/treatment-guidelines/references.htm.

[48] Muzny CA, Laniewski P, Schwebke JR, Herbst-Kralovetz MM. Host-vaginal microbiota interactions in the pathogenesis of bacterial vaginosis. Curr Opin Infect Dis. 2020;33:59–65. 31789672 10.1097/QCO.0000000000000620 PMC7265982

[49] Mena KD, Gerba CP. Risk assessment of *Pseudomonas aeruginosa* in water. In: Whitacre D, editor. Reviews of environmental contamination and toxicology, vol. 201. Boston: Springer; 2009. 10.1007/978-1-4419-0032-6_3. 10.1007/978-1-4419-0032-6_3 19484589

[50] Crone S, Vives-Flórez M, Kvich L, et al. The environmental occurrence of *Pseudomonas aeruginosa*. APMIS. 2020;128(3):220–31. 10.1111/apm.13010. 31709616 10.1111/apm.13010

[51] Ibrahim D, Jabbour JF, Kanj SS. Current choices of antibiotic treatment for *Pseudomonas aeruginosa* infections. Curr Opin Infect Dis. 2020;33(6):464–73. 10.1097/QCO.0000000000000677. 33148986 10.1097/QCO.0000000000000677

[52] Bostwick DG, Woody J, Hunt C, Budd W. Antimicrobial resistance genes and modelling of treatment failure in bacterial vaginosis: clinical study of 289 symptomatic women. J Med Microbiol. 2016;65:377–86. 26887782 10.1099/jmm.0.000236

[53] Raabe VN, Shane AL. Group B Streptococcus (*Streptococcus agalactiae*). Microbiol Spectr. 2019;7(2). 10.1128/microbiolspec.GPP3-0007-2018. Accessed 23 Oct 2023. 10.1128/microbiolspec.gpp3-0007-2018 PMC6432937 30900541

