# Supplementary material for: Bacterial vaginosis (BV) and Trichomonas vaginalis (TV) co-infection, and bacterial antibiogram profile of pregnant women studied in Lagos, Nigeria
Source: BMC Womens Health. 2024 Jul 23;24:415. doi: 10.1186/s12905-024-03257-y (PMC11264389; doi:10.1186/s12905-024-03257-y)
Supplement: Supplementary file 1 — Supplementary Material 1. [file 12905_2024_3257_MOESM1_ESM.pdf]

Socio-demographic characteristics and other factors, with Microbial (TV & bacteria) prevalence among the pregnant women studied.

NO	Age (years)	Positive	Negative	Educational status	Positive	Negative	Occupation	Positive	Negative
MAK 1	40		-	Tertiary	+		Full time housewife	+	
MAK 2	38		-	Primary	+		Student		-
MAK 3	22		-	Primary	+		Trading	+	
MAK 4	25	+		Secondary		-	Trading	+	
MAK 5	31		-	Secondary		-	Trading	+	
MAK 6	40		-	Secondary		-	Trading		-
MAK 7	19	+		Primary		-	Public servant		-
MAK 8	24		-	Secondary	+		Trading	+	
MAK 9	28	+		Secondary		-	Trading		-
MAK 10	34	+		Secondary		-	Trading	+	
MAK 11	41		-	Tertiary	+		Full time housewife		-
MAK 12	22	+		Primary		-	Trading		-
MAK 13	26		-	Secondary	+		Trading		-
MAK 14	23	+		Primary		-	Trading	+	
MAK 15	33		-	Secondary		-	Trading		-
MAK 16	36		-	Tertiary		-	Full time housewife	+	
MAK 17	25		-	Secondary		-	Trading		-
MAK 18	34	+		Secondary	+		Trading		-
MAK 19	29		-	Secondary	+		Trading	+	
MAK 20	26	+		Secondary		-	Trading	+	
MAK 21	37		-	Primary	+		Student		-
MAK 22	25		-	Secondary		-	Trading	+	
MAK 23	40		-	Tertiary	+		Full time housewife		-
MAK 24	26	+		Secondary		-	Trading	+	
MAK 25	33	+		Secondary		-	Trading		-
MAK 26	29		-	Secondary		-	Trading		-
MAK 27	30	+		Secondary		-	Trading		-
MAK 28	24		-	Primary	+		Trading	+	
MAK 29	44		-	Tertiary	+		Full time housewife	+	
MAK 30	33	+		Secondary		-	Trading		-
MAK 31	22		-	Primary	+		Trading		-
MAK32	27	+		Secondary	+		Trading		-
MAK 33	32	+		Secondary	+		Trading	+	

MAK 34	37		-	Primary	+		Student		-
MAK 35	24	+		Secondary		-	Trading		-
MAK 36	34		-	Secondary		-	Trading	+	
MAK 37	28		-	Secondary	+		Trading	+	
MAK 38	36		-	Tertiary		-	Full time housewife		-
MAK 39	32	+		Secondary	+		Trading	+	
MAK 40	21	+		Primary		-	Trading		-
MAK 41	37		-	Tertiary		-	Student		-
MAK 42	34	+		Secondary		-	Trading		-
MAK 43	40		-	Tertiary	+		Full time housewife	+	
MAK 44	22		-	Primary		-	Trading		-
MAK 45	28		-	Secondary		-	Trading		-
MAK 46	34	+		Secondary	+		Trading	+	
MAK 47	24	+		Secondary	+		Trading		-
MAK 48	39		-	Primary		-	Public servant		-
MAK 49	27		-	Secondary		-	Trading		-
MAK 50	32	+		Secondary		-	Trading		-
MAK 51	43		-	Tertiary	+		Full time housewife	+	
MAK 52	20	+		Primary	+		Trading	+	
MAK 53	28	+		Secondary	+		Trading		-
MAK 54	34	+		Secondary		-	Trading	+	
MAK 55	39		-	Primary		-	Public servant		-
MAK 56	26		-	Secondary	+		Trading		-
MAK 57	30	+		Secondary		-	Trading		-
MAK 58	34		-	Secondary	+		Trading	+	
MAK 59	36		-	Tertiary	+		Student		-
MAK 60	24		-	Secondary	+		Trading		-
MAK 61	28	+		Secondary		-	Trading		-
MAK 62	35		-	Secondary		-	Trading	+	
MAK 63	36		-	Tertiary	+		Full time housewife		-
MAK 64	23	+		Primary		-	Trading		-
MAK 65	26		-	Secondary	+		Trading	+	
MAK 66	34	+		Secondary		-	Trading	+	
MAK 67	36		-	Tertiary	+		Student		-

MAK 68	23		-	Primary		-	Trading	+	
MAK 69	27		-	Secondary		-	Trading		-
MAK 70	33	+		Secondary		-	Trading		-
MAK 71	42		-	Secondary	+		Trading	+	
MAK 72	37		-	Secondary	+		Public servant	+	
MAK 73	24	+		Primary	+		Trading	+	
MAK 74	25		-	Secondary		-	Trading	+	
MAK 75	26		-	Secondary	+		Trading	+	
MAK 76	27		-	Secondary	+		Trading	+	
MAK 77	28		-	Secondary		-	Trading		-
MAK 78	29	+		Secondary		-	Trading		-
MAK 79	30		-	Secondary		-	Trading		-
MAK 80	31		-	Secondary		-	Trading		-
MAK 81	32	+		Secondary	+		Trading		-
MAK 82	33		-	Secondary		-	Trading	+	
MAK 83	35		-	Secondary		-	Trading		-
MAK 84	20		-	Primary	+		Public servant		-
MAK 85	22	+		Primary		-	Trading		-
MAK 86	24	+		Primary	+		Trading	+	
MAK 87	27		-	Secondary		-	Trading		-
MAK 88	29		-	Secondary		-	Trading		-
MAK 89	30		-	Secondary		-	Trading		-
MAK 90	30		-	Secondary		-	Trading		-
MAK 91	31	+		Secondary	+		Trading	+	
MAK 92	33		-	Secondary		-	Trading	+	
MAK 93	35		-	Secondary		-	Trading		-
MAK 94	20	+		Primary		-	Trading		-
MAK 95	22		-	Primary	+		Trading		-
MAK 96	24		-	Secondary	+		Trading		-
MAK 97	25		-	Secondary		-	Trading		-
MAK 98	27	+		Secondary		-	Trading		-
MAK 99	30	+		Secondary		-	Trading		-
MAK 100	31		-	Secondary		-	Trading		-
MAK 101	32		-	Secondary		-	Trading	+	

MAK 102	35		-	Secondary	+		Trading		-
MAK 103	37		-	Primary	+		Student		-
MAK 104	20		-	Primary	+		Public servant	+	
MAK 105	22	+		Primary		-	Trading		-
MAK 106	24	+		Primary		-	Trading		-
MAK 107	25		-	Secondary	+		Trading		-
MAK 108	26		-	Secondary		-	Trading		-
MAK 109	29		-	Secondary		-	Trading	+	
MAK 110	30		-	Secondary		-	Trading		-
MAK 111	30	+		Secondary		-	Trading	+	
MAK 112	31	+		Secondary		-	Trading		-
MAK 113	32		-	Secondary		-	Trading		-
MAK 114	33		-	Secondary		-	Trading		-
MAK 115	34		-	Secondary	+		Trading		-
MAK 116	35	+		Secondary	+		Trading	+	
MAK 117	38		-	Primary		-	Student		-
MAK 118	21	+		Primary		-	Trading		-
MAK 119	25	+		Secondary		-	Trading	+	
MAK 120	26		-	Secondary		-	Trading	+	
MAK 121	27	+		Secondary		-	Trading		-
MAK 122	28		-	Secondary	+		Trading		-
MAK 123	29	+		Secondary		-	Trading		-
MAK 124	30		-	Secondary		-	Trading		-
MAK 125	31	+		Secondary		-	Trading		-
MAK 126	31		-	Secondary		-	Trading	+	
MAK 127	32	+		Secondary		-	Trading		-
MAK 128	33		-	Secondary	+		Trading	+	
MAK 129	37		-	Primary		-	Student	+	
MAK 130	20		-	Secondary		-	Trading		-
MAK 131	22		-	Secondary		-	Trading		-
MAK 132	24		-	Secondary	+		Trading		-
MAK 133	26		-	Secondary		-	Trading		-
MAK 134	27		-	Secondary		-	Trading		-
MAK 135	28	+		Secondary	+		Trading		-

MAK 136	27		-	Secondary		-	Trading		-
MAK 137	29		-	Secondary		-	Trading		-
MAK 138	27		-	Secondary		-	Trading	+	
MAK 139	37		-	Tertiary		-	Student		-
MAK 140	24	+		Secondary		-	Trading		-
MAK 141	19	+		Secondary		-	Public servant	+	
MAK 142	25		-	Secondary		-	Trading		-
MAK 143	28		-	Secondary		-	Trading		-
MAK 144	22		-	Primary	+		Trading		-
MAK 145	19		-	Secondary		-	Public servant	+	
MAK 146	22	+		Secondary		-	Trading		-
MAK 147	23	+		Secondary		-	Trading		-
MAK 148	25		-	Primary		-	Trading		-
MAK 149	26		-	Secondary		-	Trading		-
MAK 150	27		-	Secondary	+		Trading	+	
MAK 151	28	+		Secondary	+		Trading		-
MAK 152	36		-	Primary		-	Student		-
MAK 153	18		-	Secondary		-	Public servant		-
MAK 154	25		-	Secondary		-	Trading		-
MAK 155	26		-	Secondary		-	Trading		-
MAK 156	23		-	Primary		-	Trading		-
MAK 157	27	+		Secondary		-	Trading		-
MAK 158	28		-	Secondary		-	Trading		-
MAK 159	29	-		Secondary	+		Trading	-	
MAK 160	29		-	Secondary		-	Trading		-
MAK 161	30		-	Secondary		-	Trading		-
MAK 162	31		-	Secondary		-	Trading		-
MAK 163	32		-	Secondary		-	Trading		-
MAK 164	38		-	Primary		-	Student		-
MAK 165	21	+		Primary		-	Trading		-
MAK 166	23		-	Primary	+		Trading	+	
MAK 167	25	+		Secondary		-	Trading	+	
MAK 168	26		-	Secondary		-	Trading		-
MAK 169	28		-	Secondary		-	Trading		-

MAK 170	29		-	Secondary		-	Trading		-
MAK 171	30		-	Secondary		-	Trading		-
MAK 172	26	+		Secondary		-	Trading		-
MAK 173	31		-	Secondary	+		Trading		-
MAK 174	32		-	Secondary		-	Trading	+	
MAK 175	18	+		Primary	+		Public servant	+	
MAK 176	22	+		Primary	+		Trading	+	
MAK 177	23		-	Primary	+		Trading	+	
MAK 178	21		-	Primary		-	Trading		-
MAK 179	25		-	Secondary		-	Trading		-
MAK 180	26		-	Secondary		-	Trading		-
MAK 181	27		-	Secondary		-	Trading		-
MAK 182	29	+		Secondary	+		Trading		-
MAK 183	29		-	Secondary		-	Trading	+	
MAK 184	38		-	Primary		-	Student		-
MAK 185	21		-	Primary		-	Trading	+	
MAK 186	25	+		Secondary		-	Trading	+	
MAK 187	23	+		Primary		-	Trading		-
MAK 188	26	+		Secondary		-	Trading		-
MAK 189	24	+		Secondary	+		Trading		-
MAK 190	30		-	Secondary	+		Trading		-
MAK 191	35	+		Secondary		-	Trading	+	
MAK 192	31	+		Secondary		-	Trading	+	
MAK 193	33		-	Secondary		-	Trading	+	
MAK 194	32		-	Secondary	+		Trading		-
MAK 195	38		-	Primary	+		Public servant		-
MAK 196	24		-	Secondary	+		Trading		-
MAK 197	39		-	Primary		-	Public servant	+	
MAK 198	23	+		Primary		-	Trading		-
MAK 199	26	+		Secondary	+		Trading	+	
MAK 200	27	+		Secondary		-	Trading	+	
MAK 201	21		-	Primary	+		Trading	+	
MAK 202	28		-	Secondary	+		Trading		-
MAK 203	29	+		Secondary		-	Trading		-

MAK 204	28		-	Secondary		-	Trading		-
MAK 205	27		-	Secondary		-	Trading		-
MAK 206	27		-	Secondary	+		Trading		-
MAK 207	21		-	Primary	+		Trading	+	
MAK 208	27		-	Secondary		-	Trading	+	
MAK 209	28	+		Secondary		-	Trading	+	
MAK 210	23	+		Primary		-	Trading	+	
MAK 211	28	+		Secondary		-	Trading		-
MAK 212	29		-	Secondary		-	Trading		-
MAK 213	32		-	Secondary	+		Trading	+	
MAK 214	30		-	Secondary		-	Trading		-
MAK 215	33		-	Secondary	+		Trading	+	
MAK 216	31		-	Secondary		-	Trading	+	
MAK 217	28		-	Secondary		-	Trading		-
MAK 218	32		-	Secondary		-	Trading		-
MAK 219	36		-	Tertiary	+		Full time housewife		-
MAK 220	21	+		Primary	+		Trading		-
MAK 221	27	+		Secondary		-	Trading	+	
MAK 222	29		-	Secondary		-	Trading		-
MAK 223	23		-	Primary	+		Trading		-
MAK 224	26		-	Secondary	+		Trading		-
MAK 225	30		-	Secondary	+		Trading		-
MAK 226	35		-	Secondary		-	Trading		-
MAK 227	25	+		Secondary		-	Trading		-
MAK 228	33	+		Secondary		-	Trading	+	
MAK 229	23	+		Primary		-	Trading	+	
MAK 230	31		-	Secondary		-	Trading	+	
MAK 231	27		-	Secondary		-	Trading		-
MAK 232	28	+		Secondary	+		Trading		-

Marital status	Positive	Negative	Number oF sex partners	Positive	Negative	Gestation period	Postive	Negative
Polygamy	+		Two or more partners	+		Undeclared	+	
Polygamy	+		Two or more partners	+		Undeclared	+	
Monogamy	+		Declined response		-	First trimester		-
Monogamy		-	One sex partner		-	Second trimester	+	
Monogamy		-	One sex partner		-	Third trimester		-
Monogamy	+		Two or more partners	+		Third trimester	+	
Polygamy	+		Declined response		-	First trimester	+	
Monogamy	+		One sex partner		-	Second trimester		-
Monogamy		-	One sex partner	+		Third trimester	+	
Monogamy	+		One sex partner		-	Third trimester		-
Polygamy	+		Two or more partners	+		Undeclared		-
Monogamy		-	Declined response		-	First trimester		-
Monogamy	+		One sex partner	+		Second trimester		-
Monogamy		-	Declined response	+		First trimester	+	
Monogamy		-	One sex partner	+		Third trimester		-
Polygamy	+		Two or more partners	+		Undeclared		-
Monogamy		-	One sex partner		-	Second trimester	+	
Monogamy	+		One sex partner		-	Third trimester	+	
Monogamy	+		One sex partner	+		Third trimester		-
Monogamy	+		One sex partner		-	Second trimester	+	
Polygamy	+		Two or more partners	+		Undeclared		-
Monogamy		-	One sex partner	+		Second trimester		-
Polygamy	+		Two or more partners	+		Undeclared		-
Monogamy		-	One sex partner		-	Second trimester	+	
Monogamy	+		One sex partner	+		Third trimester	+	
Monogamy	+		One sex partner		-	Third trimester		-
Monogamy		-	One sex partner		-	Third trimester	+	
Monogamy	+		Declined response		-	First trimester		-
Polygamy	+		Two or more partners	+		Undeclared		-
Monogamy	+		One sex partner		-	Third trimester		-
Monogamy	+		Declined response		-	First trimester	+	
Monogamy		-	One sex partner	+		Third trimester		-
Monogamy		-	One sex partner	+		Third trimester	+	

Polygamy	+		Two or more partners	+		Undeclared	+	
Monogamy		-	One sex partner		-	Second trimester	+	
Monogamy	+		One sex partner		-	Third trimester	+	
Monogamy	+		One sex partner	+		Third trimester		-
Polygamy	+		Two or more partners	+		Undeclared		-
Monogamy		-	One sex partner	+		Third trimester		-
Monogamy	+		Declined response		-	First trimester	+	
Polygamy	+		Two or more partners	+		Undeclared		-
Monogamy		-	One sex partner		-	Third trimester		-
Polygamy	+		Two or more partners	+		Undeclared	+	
Monogamy		-	Declined response	+		First trimester	+	
Monogamy		-	One sex partner		-	Third trimester	+	
Monogamy		-	One sex partner		-	Third trimester		-
Monogamy	+		One sex partner		-	Second trimester	+	
Polygamy	+		Two or more partners	+		First trimester	+	
Monogamy		-	One sex partner		-	Second trimester		-
Monogamy		-	One sex partner		-	Third trimester		-
Polygamy	+		Two or more partners	+		Undeclared		-
Polygamy	+		Declined response		-	First trimester		-
Monogamy	+		One sex partner	+		Third trimester	+	
Monogamy		-	One sex partner	+		Third trimester		-
Polygamy	+		Two or more partners	+		First trimester	+	
Monogamy		-	One sex partner		-	Second trimester	+	
Monogamy		-	One sex partner		-	Third trimester		-
Monogamy	+		One sex partner	+		Third trimester	+	
Polygamy	+		Two or more partners	+		Undeclared		-
Monogamy	+		One sex partner		-	Second trimester	+	
Monogamy		-	One sex partner	+		Third trimester		-
Monogamy		-	One sex partner	+		Third trimester		-
Polygamy	+		Two or more partners	+		Undeclared		-
Monogamy		-	Declined response		-	First trimester		-
Monogamy		-	One sex partner		-	Second trimester	+	
Monogamy		-	One sex partner	+		Third trimester		-
Polygamy	+		Two or more partners	+		Undeclared		-

Monogamy	+		Declined response		-	First trimester	+	
Monogamy		-	One sex partner		-	Third trimester		-
Monogamy		-	One sex partner		-	Third trimester	+	
Monogamy	+		One sex partner	+		Third trimester	+	
Monogamy		-	One sex partner		-	Third trimester		-
Polygamy	+		One sex partner		-	Second trimester		-
Monogamy		-	One sex partner		-	Third trimester		-
Monogamy		-	One sex partner		-	First trimester	+	
Monogamy		-	Declined response		-	Third trimester		-
Monogamy		-	One sex partner	+		Third trimester		-
Monogamy		-	One sex partner		-	Third trimester		-
Monogamy	+		One sex partner		-	Third trimester	+	
Monogamy		-	One sex partner		-	Third trimester	+	
Monogamy		-	One sex partner	+		Third trimester		-
Monogamy		-	One sex partner		-	Third trimester		-
Monogamy	+		One sex partner		-	Third trimester		-
Polygamy	+		Declined response	+		First trimester		-
Monogamy		-	Declined response	+		First trimester	+	
Monogamy		-	One sex partner	+		First trimester		-
Monogamy		-	One sex partner		-	Third trimester		-
Monogamy		-	One sex partner	+		Third trimester	+	
Monogamy		-	One sex partner		-	Third trimester		-
Monogamy	+		One sex partner		-	Third trimester	+	
Monogamy		-	One sex partner	+		Third trimester		-
Monogamy		-	One sex partner		-	Third trimester		-
Monogamy	+		One sex partner		-	Third trimester		-
Monogamy		-	Declined response		-	First trimester		-
Monogamy		-	Declined response		-	First trimester	+	
Monogamy		-	One sex partner		-	Second trimester		-
Monogamy		-	One sex partner		-	Second trimester		-
Monogamy		-	One sex partner	+		Third trimester		-
Monogamy	+		One sex partner	+		Third trimester	+	
Monogamy		-	One sex partner		-	Third trimester		-
Monogamy		-	One sex partner		-	Third trimester		-

Monogamy	-	One sex partner	-	Third trimester	-
Polygamy	+	Two or more partners	+	Undeclared	-
Polygamy	+	Declined response	-	First trimester	-
Monogamy	+	Declined response	-	First trimester	-
Monogamy	-	One sex partner	-	First trimester	-
Monogamy	-	One sex partner	-	Second trimester	+
Monogamy	-	One sex partner	-	Second trimester	-
Monogamy	-	One sex partner	-	Third trimester	-
Monogamy	-	One sex partner	+	Third trimester	-
Monogamy	+	One sex partner	+	Third trimester	+
Monogamy	-	One sex partner	+	Third trimester	-
Monogamy	-	One sex partner	-	Third trimester	+
Monogamy	-	One sex partner	-	Third trimester	+
Monogamy	-	One sex partner	-	Third trimester	-
Monogamy	-	One sex partner	+	Third trimester	-
Polygamy	+	Two or more partners	+	Undeclared	+
Monogamy	-	Declined response	-	First trimester	-
Monogamy	-	One sex partner	+	Second trimester	-
Monogamy	-	One sex partner	+	Second trimester	-
Monogamy	-	One sex partner	+	Third trimester	-
Monogamy	-	One sex partner	-	Third trimester	-
Monogamy	-	One sex partner	-	Third trimester	+
Monogamy	-	One sex partner	-	Third trimester	+
Monogamy	-	One sex partner	-	Third trimester	+
Monogamy	-	One sex partner	-	Third trimester	-
Monogamy	+	One sex partner	+	Third trimester	-
Polygamy	+	One sex partner	-	Third trimester	-
Monogamy	-	One sex partner	-	Second trimester	-
Monogamy	-	Two or more partners	+	Third trimester	-
Monogamy	-	One sex partner	-	Third trimester	+
Monogamy	-	One sex partner	-	Third trimester	+
Monogamy	-	One sex partner	-	Third trimester	-
Monogamy	-	Declined response	+	Third trimester	-
Monogamy	+	One sex partner	-	Second trimester	-

Monogamy	-	One sex partner	-	Third trimester	-
Monogamy	-	One sex partner	-	Third trimester	-
Monogamy	-	One sex partner	+	Second trimester	-
Polygamy	+	Two or more partners	+	Undeclared	-
Monogamy	-	One sex partner	-	First trimester	-
Polygamy	+	Declined response	-	First trimester	+
Monogamy	-	One sex partner	-	Second trimester	+
Monogamy	-	One sex partner	-	Third trimester	-
Monogamy	-	Declined response	+	First trimester	-
Polygamy	+	Declined response	+	First trimester	-
Monogamy	-	Declined response	-	First trimester	+
Monogamy	-	One sex partner	-	First trimester	+
Monogamy	-	One sex partner	-	Second trimester	+
Monogamy	-	One sex partner	-	Second trimester	-
Monogamy	-	One sex partner	-	Second trimester	-
Monogamy	-	One sex partner	+	Third trimester	+
Polygamy	+	Two or more partners	+	Undeclared	-
Polygamy	+	Declined response	-	First trimester	-
Monogamy	-	One sex partner	-	Second trimester	-
Monogamy	-	One sex partner	-	Second trimester	+
Monogamy	-	Declined response	-	First trimester	+
Monogamy	-	One sex partner	-	Second trimester	-
Monogamy	-	One sex partner	-	Third trimester	-
Monogamy		One sex partner	-	Third trimester	-
Monogamy	-	One sex partner	+	Third trimester	-
Monogamy	-	One sex partner	+	Third trimester	-
Monogamy	-	One sex partner	-	Third trimester	-
Monogamy	-	One sex partner	-	Third trimester	+
Polygamy	+	Two or more partners	+	Undeclared	-
Monogamy	-	Declined response	+	First trimester	+
Monogamy	-	Declined response	-	First trimester	-
Monogamy	+	One sex partner	+	Second trimester	-
Monogamy	-	One sex partner	+	Second trimester	+
Monogamy	-	One sex partner	+	Third trimester	-

Monogamy	-	One sex partner	-	Third trimester	-
Monogamy	-	One sex partner	-	Second trimester	+
Monogamy	-	One sex partner	-	Third trimester	-
Monogamy	-	One sex partner	-	Third trimester	-
Monogamy	+	One sex partner	-	Third trimester	-
Polygamy	+	Declined response	+	First trimester	+
Monogamy	-	Declined response	-	First trimester	+
Monogamy	-	Declined response	+	First trimester	-
Monogamy	-	Declined response	-	First trimester	-
Monogamy	-	One sex partner	-	Second trimester	-
Monogamy	+	One sex partner	+	Second trimester	-
Monogamy	-	One sex partner	-	Second trimester	-
Monogamy	-	One sex partner	+	Third trimester	+
Monogamy	+	One sex partner	+	Third trimester	+
Polygamy	+	Two or more partners	+	Undeclared	-
Monogamy	-	Declined response	-	First trimester	-
Monogamy	-	One sex partner	-	Second trimester	-
Monogamy	-	Declined response	+	First trimester	+
Monogamy	-	One sex partner	-	Second trimester	+
Monogamy	-	One sex partner	-	Second trimester	-
Monogamy	+	One sex partner	-	Third trimester	-
Monogamy	+	One sex partner	+	Third trimester	-
Monogamy	+	One sex partner	-	Third trimester	-
Monogamy	-	One sex partner	-	Third trimester	-
Monogamy	-	One sex partner	+	Third trimester	+
Polygamy	+	Two or more partners	+	First trimester	+
Monogamy	-	One sex partner	-	Second trimester	-
Polygamy	+	Two or more partners	+	First trimester	+
Monogamy	+	Declined response	-	First trimester	-
Monogamy	+	One sex partner	+	Second trimester	-
Monogamy	-	One sex partner	+	Third trimester	-
Monogamy	-	Declined response	+	First trimester	-
Monogamy	-	One sex partner	-	Third trimester	-
Monogamy	-	One sex partner	-	Third trimester	-

Monogamy		-	One sex partner		-	Third trimester		-
Monogamy	+		One sex partner		-	Second trimester	+	
Monogamy		-	One sex partner	+		Third trimester	+	
Monogamy		-	Declined response	+		First trimester		-
Monogamy		-	One sex partner		-	Second trimester		-
Monogamy		-	One sex partner		-	Third trimester		-
Monogamy	+		Declined response		-	First trimester		-
Monogamy	+		One sex partner		-	Third trimester		-
Monogamy	+		One sex partner		-	Third trimester		-
Monogamy		-	One sex partner	+		Third trimester	+	
Monogamy		-	One sex partner		-	Third trimester		-
Monogamy	+		One sex partner		-	Third trimester		-
Monogamy		-	One sex partner		-	Third trimester		-
Monogamy		-	One sex partner	+		Third trimester		-
Monogamy		-	One sex partner		-	Third trimester		-
Polygamy	+		Two or more partners	+		Undeclared	+	
Monogamy		-	Declined response		-	First trimester	+	
Monogamy		-	One sex partner		-	Second trimester	+	
Monogamy	+		One sex partner		-	Third trimester		-
Monogamy	+		Declined response		-	First trimester	+	
Monogamy	+		One sex partner		-	Second trimester		-
Monogamy		-	One sex partner	+		Third trimester		-
Monogamy		-	One sex partner	+		Third trimester		-
Monogamy		-	One sex partner	+		Second trimester		-
Monogamy		-	One sex partner		-	Third trimester		-
Monogamy		-	Declined response		-	First trimester	+	
Monogamy	+		One sex partner		-	Third trimester		-
Monogamy		-	One sex partner		-	Second trimester		-
Monogamy		-	One sex partner	+		Third trimester		-
